# Stochastic modelling of tyrosine kinase inhibitor rotation therapy in chronic myeloid leukaemia

**DOI:** 10.1186/s12885-019-5690-5

**Published:** 2019-05-28

**Authors:** H. Jonathan G. Lindström, Astrid S. de Wijn, Ran Friedman

**Affiliations:** 1 Department of Chemistry and Biomedical Sciences, Linnæus University, Kalmar, 391 82 Sweden; 2 Department of Mechanical and Industrial Engineering, Norwegian University of Science and Technology, Trondheim, 7491 Norway

**Keywords:** Chronic myeloid leukaemia, Drug rotation, Treatment Simulation, Targeted therapy, Stochastic modelling

## Abstract

**Background:**

Resistance towards targeted cancer treatments caused by single nucleotide variations is a major issue in many malignancies. Currently, there are a number of available drugs for chronic myeloid leukaemia (CML), which are overcome by different sets of mutations. The main aim of this study was to explore if it can be possible to exploit this and create a treatment protocol that outperforms each drug on its own.

**Methods:**

We present a computer program to test different treatment protocols against CML, based on available resistance mutation growth data. The evolution of a relatively stable pool of cancer stem cells is modelled as a stochastic process, with the growth of cells expressing a tumourigenic protein (here, Abl1) and any emerging mutants determined principally by the drugs used in the therapy.

**Results:**

There can be some benefit to Bosutinib-Ponatinib rotation therapy even if the mutation status is unknown, whereas Imatinib-Nilotinib rotation is unlikely to improve the outcomes. Furthermore, an interplay between growth inhibition and selection effects generates a non-linear relationship between drug doses and the risk of developing resistance.

**Conclusions:**

Drug rotation therapy might be able to delay the onset of resistance in CML patients without costly ongoing observation of mutation status. Moreover, the simulations give credence to the suggestion that lower drug concentrations may achieve better results following major molecular response in CML.

**Electronic supplementary material:**

The online version of this article (10.1186/s12885-019-5690-5) contains supplementary material, which is available to authorized users.

## Background

Targeted therapies, which directly target molecular pathways critical to tumours instead of rapidly dividing cells in general, have in many cases improved survival significantly when compared to cytotoxic chemotherapy or radiation. Unfortunately, a recurring difficulty with targeted therapies is the occurrence of resistance [1]. As these therapies target oncogenic molecular pathways with high specificity, smaller, relatively common changes such as mutations in the molecular drug target, activation of alternate pathways and overexpression of the drug target or of transporter proteins, can render them ineffective [2]. Even if such a change occurs in a single cancer-cell it confers a fitness advantage that can generate a cell lineage which reproductively outpaces the rest of the tumour. In competition with the other cells, this gives such a lineage a higher probability of becoming a major fraction of the tumour cells [3]. This clonal evolution among the tumour cells allows major and minor resistance-giving traits to propagate in the population [4]. Once a large proportion of the tumour becomes resistant, the success of continued treatment is unlikely. In many cases, the fitness advantage which allows resistant clones to expand exists only during treatment. Thus, by altering the treatment protocol, it is possible that we could steer evolution towards a treatable state [1, 5]. The vast number of possible protocols limit our ability to explore this in experiments. On the other hand, theoretical studies and computer modelling allow testing on an otherwise infeasible scale [6] and thus open a venue for estimation of multiple treatment protocols [7].

One of the first malignancies where targeted therapies proved effective was chronic myeloid leukaemia (CML) that involves the constitutively active tyrosine kinase Abl1. In most cases of CML, a chromosomal translocation creates Bcr-Abl, a fusion protein in which the Abelson tyrosine kinase (Abl) is stripped of its regulatory regions, leaving the kinase domain free to catalyse any substrate it comes across, triggering numerous growth pathways as a result [8]. It has been shown that Bcr-Abl is sufficient for producing the malignant phenotype on its own. As the protein is both the sole driver and absent in healthy cells it is also an excellent drug target [9]. Imatinib, a Bcr-Abl specific tyrosine kinase inhibitor (TKI), was approved in 2001 and has become the standard CML treatment due to greatly improved outcomes over traditional therapy [10]. As it inhibits Bcr-Abl it effectively shuts down the signalling responsible for creating the malignant phenotype. In spite of this, Imatinib and related TKIs rarely cure CML. Instead, a minimal residual disease remains and most often patients continue treatment for the rest of their lives to prevent recurrence. This is thought to be caused by Bcr-Abl independent cancer stem cells, which carry the gene but do not require it [9].

A significant portion of Imatinib treatment failures are due to mutations which emerge in the kinase domain of Bcr-Abl that reduce the efficiency of Imatinib. To remedy this, a series of other TKIs have been developed that do not share the same resistance mutations and have an overall higher affinity for Bcr-Abl. These are most often used as a second-line treatment. For instance, the single nucleotide variations (SNVs) E255V and Y253H confer resistance towards Imatinib but not Dasatinib or Bosutinib, respectively [11]. Until recently T315I was the only untreatable Abl1-SNV, because the mutated protein was resistant towards all available drugs. The Bcr-Abl inhibitor Ponatinib approved in 2012 has since changed that but is associated with more severe side effects than other TKIs, most notably vascular occlusion events, heart failure, and hepatotoxicity [12]. The EPIC trial comparing Ponatinib to Imatinib was terminated early due to arterial thrombotic events in some patients [13]. Thus, despite being less vulnerable to resistance mutations, Ponatinib is only given when no other options are available. Moreover, two mutations in the same copy of Abl1 (compound mutations) can cause resistance towards Ponatinib [14], and some SNVs, such as G250E and E255V, yield some degree of Ponatinib resistance (at least in vitro) [11].

The sensitivity of known mutants towards approved and experimental drugs was the subject of several studies (e.g., [11, 15, 16]). The results are often presented as IC_50_-values, i.e., the TKI concentration at which cell proliferation is slowed by 50%. Mutations can change the drug binding protein such that the affinity for the drug is reduced, resulting in less effective inhibition. These data are a proxy for the fitness of individual mutants under treatment, and are used as such in this work.

**Fig. 1:**
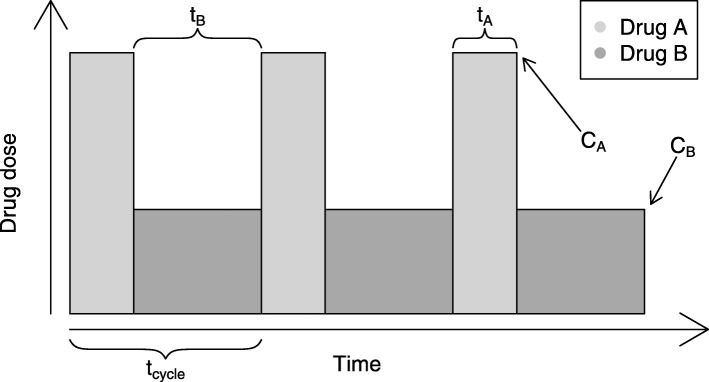
Example of a drug rotation protocol. The protocol is defined by four variables: The drug doses (*C*_*A*_, *C*_*B*_), and the time per cycle (*t*_*A*_+*t*_*B*_=*t*_*cycle*_) of either drug

The type of non-trivial protocols investigated in this paper are primarily drug rotations, where drugs are switched according to a fixed pattern as illustrated in Fig. 1. A range of both doses and timings were investigated. The drug rotations investigated maintained a constant drug pressure on the tumour, much like conventional treatments; but the source of this pressure changes over time. This is in contrast to the conventional treatment model, where a patient receives the same drug as long as the drug is tolerated and effective, and is moved to a new drug (if available) otherwise. With Imatinib the dose can sometimes be reduced after an initial effect has been established [17]. It is however uncommon to terminate Imatinib treatment. Termination of treatment when active stem cells are still present is liable to cause a quick return of symptoms and increases the risk of disease progression, possibly via accumulating secondary adaptations, into accelerated phase or blast crisis with significantly worse treatment outcomes. Even with newer TKIs, there are as of yet no clear guidelines on the possibility of seceding treatment in CML [18].

On top of the potential benefits with respect to drug resistance, a drug rotation can help manage side effects, which is especially pertinent for Ponatinib, while also lowering the overall risk of resistance. If the two drugs have little or no overlapping resistance mechanisms the likelihood of adapting to both may be lower, while singly resistant cells are still exposed to one drug towards which they are sensitive. However, there is also a risk for development of mutations that make the molecular drug target resistant to both drugs. In principle, combination therapies where several molecular targets are treated at the same time are beneficial in this aspect [19], but such therapies are not currently available for CML. Using multiple Bcr-Abl TKIs simultaneously means they have to compete with one another, as they all target the same binding pocket of the enzyme. Thus such combination therapy is expected to be less efficient in eradicating the cancer cells. Because of these factors it is unlikely that combinations would allow for significant dose reductions, and because of side-effect concerns in giving multiple TKIs simultaneously we decided not to pursue it in this work.

In this paper, we investigate whether drug rotation protocols using TKIs for the treatment of CML could reduce the risk of resistance. It is shown that drug rotations with Ponatinib have the largest potential benefits. Furthermore, the risk of developing resistance seems tied to the achieved degree of inhibition with reduced risks for both low (<40%) and high (>90%) degrees of inhibition.

## Methods

### Modelling approach and assumptions

If the growth rate of any particular mutant subject to treatment is known, it becomes possible to simulate the evolution of CML cells under a time-varying treatment protocol. Since cell growth and mutation are not entirely deterministic, the evolution of CML cells is appropriately modelled as a stochastic process. The derivation of such a model enables the investigation of potentially superior non-trivial treatment protocols for CML.

The branching process [20] models cell growth as a stochastic process where individual cells at the end of their life give birth to (possibly mutated) offspring according to some probability distribution. Branching processes have previously been used to (among many examples): optimise screening in ovarian cancer [21], evaluate the effects of combination therapy [19] and numerous other modelling instances of treatment response and resistance prevention [22]. We propose a model similar to the standard branching process, albeit slightly restructured for more convenient computer simulation. Furthermore, we consider only a non-hierarchical, stable number of cancer cells (but with fluctuations about some mean) capable of self replication (cancer stem cells, CSCs). CSCs are important in modelling of cancers [23] and in particular in CML [24]. It should be noted that in cells that do not self-replicate (i.e., they are not CSCs) there can be no lasting adaptations, as their lineage is bound to die and no traits can be permanently acquired. If the CSCs replicate in a strict hierarchy the population capable of sustaining a mutation is further reduced [25]. The CSC pool also grows more slowly than differentiated cells [24] and the cells are considerably less sensitive to Abl1 inhibitors [26], motivating our choice to model the CSC pool as stable even under treatment. To that end, we introduce an adaptive death rate which means that the model proposed here is not equivalent to a typical branching process. In the context of this model, we judge resistance progression by the makeup of the CSC pool. A common assumption is that CML CSCs are entirely insensitive to TKIs [24, 27]; However, experimental studies show that while TKIs cannot kill CSCs, they have some degree of antiproliferative effect [28, 29], a phenomenon that our model replicates.

### Computational model

The discrete time model of stochastic cell growth proceeds essentially as follows. Initially, a population of cells is set up. A simple two-step procedure is then repeated iteratively: (each step will be described in further detail below)

1. Each cell has a chance to die.
2. Each of the surviving cells has a chance to reproduce, with a small probability of producing a mutant.

Treatment protocols are simulated by letting the reproduction probability change with time in a way that depends on the mutation status of the cell. A pseudocode description of the algorithm is available in Section 8 of Additional file 1.

In every iteration, there is some probability *q* for each cell to die. The cells that survive reproduce with a probability *s*_*i*_ that depends on their genotype *i*. To reduce the computational burden, all cells of an identical genotype are treated together, and thus the total number of deaths or births for one group per iteration is described by the binomial distribution *B*(*n*,*p*) for *n* independent but identical cell events (i.e. births/deaths/mutations), and probability *p* (which can be birth-/death- or mutation-probability). Under the effect of a single drug, the birth-probabilities, i.e. the odds of a cell of type *i* dividing in a particular timestep (analogous to a birth rate in a continuous setting) are calculated as

1 $$ s_{i}(t) = s_{i}^{(0)} 2^{-C(t)/{IC}_{50}}   $$

where *C*(*t*) is the time-dependent drug concentration (as exemplified in Fig. 1), and the IC_50_ depends on both genotype and drug. IC_50_ values are taken from [11], where these values are available for all single mutations and some, but not all compound mutations. This use of IC_50_ values is discussed further in Additional file 1: Section 4. In cases where IC_50_ values for compound mutations are not available, they are estimated as the maximum IC_50_ of the mutation’s constituent SNVs which is a decent approximation in many cases (Additional file 1: Figure S2) when no other data exists, though it is known that some compound mutations are much more highly resistant than expected [14]. The starting birth-probabilities $s_{i}^{(0)}$ can be unified or set separately for each genotype. A method for deriving these from position specific scoring matrix (PSSM) data is provided in the Additional file 1: Section 1. However, the rate of evolution in the presence of drug therapy is in fact almost independent of the background reproduction rate of drug-free tumour cells, since the presence of drug therapy alters the evolutionary landscape to a much greater degree. Differences in the inherent growth rate are thus insignificant compared to the much larger reproduction rate differences induced by treatment. On the contrary, if periods of treatment-free growth were to be simulated, variations in reproduction rate would likely be important [30].

Under the assumptions that only cancer stem cells lead to resistant clones [24] and that these cells are rather resistant to the drugs [9] the effective population size is kept stable, though fluctuations about the mean are allowed. To keep the population size average fixed, the death-probability is rebalanced at the start of every iteration

2 $$ q(t) = {\sum_{i}N_{i} s_{i}(t) \over N}\left(1 + \kappa{N - \hat{N} \over \hat{N}}\right){N \over N + \sum_{i}N_{i} s_{i}(t)}   $$

*N* is the total population size, $\hat {N}$ is the desired mean and *κ* is a spring constant describing how strongly *N* tends towards $\hat {N}$ (Table 1). $\sum _{i}N_{i}s_{i}(t)$ is the expected number of new cells (since *E*[*B*(*n*,*p*)]=*np*). There are three components to this equation. The first factor is simply the average birth rate at that timestep which, in a deterministic setting, is what the death rate should equal for the population to remain constant. The second factor is used to increase or decrease the death rate when the population size is too high or low respectively. This causes the population size to drift towards $\hat {N}$ as shown in Figure S1 in Additional file 1; how quickly it does so depends linearly on the difference between *N* and $\hat {N}$. Finally, the last factor accounts for the drift otherwise induced by the algorithm (killing cells first and then letting them reproduce). The population size oscillates up and down as cells reproduce and are killed in turn; this factor makes sure that the population size is $\hat {N}$ on average at the start of each timestep.

**Table 1:** Global parameters

Parameter	Symbol	Value
Population size avg.	$\hat {N}$	250 000 cells [24]
Population size spring constant	*κ*	1.0
Mutation rate multiplier	*μ*	10^−7^ mutations/division^a^
Unmodified growth rate	*s* ^(0)^	0.016 divisions/timestep^b^ [30]

^a^The number of mutations in a single residue per new cell where only one transversion can result in the specific amino-acid change. Mutations that result from transitions or more than one possible SNV have higher mutation rates, see Additional file 1: Section 5 and Table S1

^b^Corresponds to approximately 0.3 – 0.4 divisions per day

Whenever a new cell is produced it has a small chance *μ*_*i*_ of being a mutant. Only single nucleotide variations (SNVs) are allowed in a single cell division (though they may accumulate and form compound mutations over time). Mutation probabilities are specified at a genome level and includes altered transition/transversion ratios (ts/tv=2). Explicitly modelling the genetic code for possible resistance mutation spots avoids some potential cases of a residue-residue transition probability matrix. This, and the multiplier *ε*_*ji*_ are explained in further detail in Section 5 in Additional file 1. Mutations that result in a variant with a known drug sensitivity are kept. Synonymous mutations are also kept. All other mutations are assumed to be deleterious or irrelevant and are not accepted. Backwards mutations that restore the wildtype from a mutant are allowed.

Taking only the leading order behaviour of growth- and death-rates into account, the average the change in population for a genotype in one iteration is

3 $$ \begin{aligned} N_{i}(t+1) &= \overbrace{[1 + s_{i}(1-\mu_{i})] {[1-q(t)]} N_{i}(t)}^{\text{Growth term}}\\ &\quad+ \underbrace{\sum_{j\in {nn}_{i}} s_{j} \mu \epsilon_{ji} N_{j}(t) {[1-q(t)]}}_{\text{Mutation term}} \end{aligned}   $$

where the sum in the mutation term is over all other genotypes that could mutate into *i* (nearest neighbours, *nn*_*i*_). Let $\mathbb {P}[\dots ]$ denote the probability of an event. Since

4 $$ \epsilon_{ji} \equiv {\mathbb{P}[j\mathrm{~mutates~to~}i] \over \mathbb{P}[\mathrm{Single~ transversion}]} = {\mathbb{P}[j\mathrm{~mutates~to~}i] \over \mu}   $$

and *μ* is a constant mutation rate that depends on the behaviour of the cancer cells (see Table 1), *με*_*ji*_ is the chance of *j* mutating to *i* specifically and

5 $$ \mu_{i} = \sum_{j\in {nn}_{i}} \mu \epsilon_{ij}   $$

is the chance of any specific residue mutating. A single transversion is chosen as the baseline since is the most unlikely type of SNV. Higher order terms appear in Eq. (3) if population size fluctuations are taken into account. When the drug concentrations are stable, the waiting time between events will effectively have a geometric distribution, which is the discrete analogue of the exponential waiting times characteristic of Poisson processes. Thus, the cells have no memory of whether they were recently changed by a mutation or not.

### Implementation

All simulations were run using the same global parameters (Table 1); $\hat {N}$ and *μ* were selected based on conditions that are plausible for a newly diagnosed patient. We assume that CML is driven by a relatively large (2.5·10^−5^ cells) CSC pool with no clearly defined internal hierarchy. If CML has a more distinct hierarchic structure, the relevant CSC pool might be vastly smaller as only the most basal (stem-cell like) cells can sustain a mutation [25, 27]. In the case of small population sizes, stochastic effects dominate the evolution [31], and effects from the treatment prototol, if at all present, will be harder to detect.

By correlating the time until Imatinib resistance would generally occur in practice [32, 33] and the time it takes for a rare mutation to grow to a major fraction of the CSC population [34] with simulations, these parameters were determined to result in timesteps that are about 1.2 h long (600 timesteps/month). Simulations were started with Bcr-Abl cells where 100% of the cells carried Abl1 with wildtype kinase domain. IC_50_ values for known resistance mutations are taken from [11]. It is not known for certain whether resistant cells exist prior to treatment [35, 36] but the results starting from a pure wildtype population are still valid subject to the condition that the resistant cells are a minor fraction of the population when treatment starts (which is essentially equivalent to removing the waiting time for mutations to occur). The effects from drug-rotations on controlling the outgrowth of extant mutations remains the same.

There are several ways to simulate drug doses explicitly. One approach is to refer to the concentration of the drugs in the plasma which is available from experimental measurements. This approach, however, is not without limitations, as the treatment effects of available drugs do not match the achieved blood plasma concentration very well [37, 38]. One mechanism for this discrepancy could be that the drugs bind to a different degree to serum proteins, as observed with other small molecule drugs [39]. For instance, TKI doses based on blood plasma concentrations incorrectly indicate that Nilotinib is superior to any other available CML drug, as its achievable plasma concentration is over 100 times its wildtype IC_50_, and high enough that it should be effective against known resistance mutations. In practice, however, there are resistance mutations that make Nilotinib inactive. Owing to these limitations, we approximate the drug doses by the percent inhibition they achieve in vivo. This cannot be directly correlated to available experimental results, but could in principle be measured in cell-lines that are harvested directly from patients.

Drug doses were modelled as constant throughout the simulations. It might be argued that a wave-form is more suitable to represent drug doses. While this is true in some cases (e.g., drugs that are given once per week or less often and are slowly absorbed, or drugs that are rapidly cleared from the body), a constant value is used here owing to the frequent medication (at least once per day) and the relatively slow clearance of most the drugs. Thus, they establish a reasonably stable dose over a few weeks and the cycle times considered here of (1-4 months) are well above that. The exception is Dasatinib with a short half life of only 4h [40] but even dasatinib is not completely eliminated from the body within a week after a single dose (data taken from the registry of Pharmaceutical Specialists in Sweden [41]).

Some software implementation details are provided in Additional file 1: Section 2.

## Results

As a general measure of when resistance occurs we define $\text {WT}_{\frac {1}{2}}$ as the number of iterations before the wildtype makes up half of the total population.

6 $$ \text{WT}_{\frac{1}{2}} \equiv \mathrm{timesteps~before~} N_{wt} < {N \over 2}   $$

Stochastic effects are most significant for low population phenotypes, and at $\text {WT}_{\frac {1}{2}}$ it is very likely that the total population consists mostly of a few highly populated phenotypes. These grow rather deterministically, which means that estimated $\text {WT}_{\frac {1}{2}}$ values do not display large stochastic fluctuations.

### TKI dose scaling effects

**Fig. 2:**
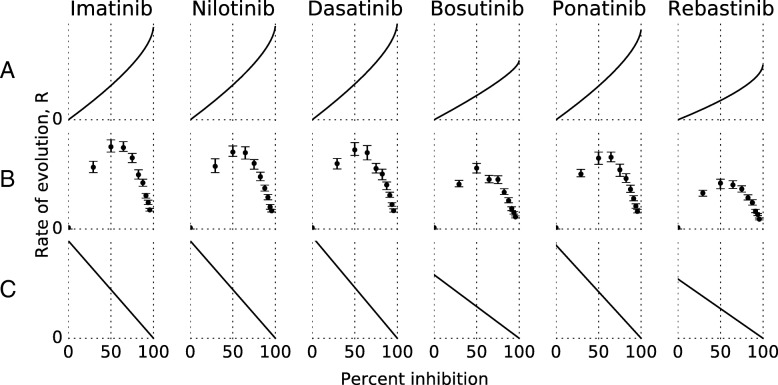
The rate of evolution varies with inhibition. The y-axis is in arbitrary units and scale is consistent within rows but not comparable between rows. **a** Pure fitness effects, with a constant reproduction rate. Derived from Moran model [42], see Eq. 7. **b** Our model. The inverse of median $\text {WT}_{\frac {1}{2}}$ from simulation with the errorbars showing a 95% confidence interval. The slightly jagged appearance comes from random variation in the stochastic simulations and from having fewer data points than in **a** or **c**. **c** Pure reproduction rate effects, i.e. simulations where any resistance mutation is guaranteed to be fixed

Figure 2 displays the rate of evolution of tumour cells as a function of percent inhibition, under different models: pure fitness effects (Moran model, Fig. 2a), our numerical model as described below (Fig. 2b), and a theoretical model, where any resistance mutation is guaranteed to be fixed (Fig. 2c, model details in Additional file 1: Section 3). In our model, simulations were run at doses that correspond to an (initial) growth rate inhibition of 29%–96% for all drugs where the required IC_50_ data could be obtained (Fig. 2b). The results were then used to examine the effects of TKI doses on the overall rate of evolution *R* i.e., the rate at which mutants become dominant in the population. It is assumed that under these conditions the most dominant population of cells will eventually become evolutionary fixed if the tumour grows for enough generations. This may or may not happen within the patient’s lifetime. For practical reasons fixation in simulations is therefore estimated using the $\text {WT}_{\frac {1}{2}}$ concept (Eq. 6). Thus, we refer to populations that have reached $\text {WT}_{\frac {1}{2}}$ as being fixed, and to the probability to reach $\text {WT}_{\frac {1}{2}}$ as the fixation probability. Explicitly, *R* is calculated as:

$$\begin{array}{*{20}l} R &= \mathrm{(near)~fixation~probability} \times \mathrm{mutation~rate}\\ &\quad\times \mathrm{reproduction~rate} \end{array} $$

Bosutinib and Rebastinib have a somewhat lower rate of evolution, due to the smaller number of known, however weak, resistance mutations. In the case of Rebastinib, this is likely because its different binding mode means the usual resistance mutations are less relevant, and as it is not clinically approved there is almost no clinical data on resistance. Of note, a similar bias may exist for other drugs (except Imatinib), as the in-vitro screening of mutants is based around mutations known primarily from Imatinib-treated patients. Ponatinib is in practice only vulnerable to compound mutations. In vitro studies reveal that many mutations confer some resistance to Ponatinib [14], but the percent inhibition that is achieved in patients is apparently enough to make the inhibitor useful anyhow. In effect, this means that a standard Ponatinib treatment results in very high percent inhibition and thus low rates of evolution. Interestingly, this does not affect the shape of the curve(s) in Fig. 2. Note that the maximum rate of evolution occurs slightly above 50% inhibition in our model (Fig. 2 row b) regardless of the inhibitor. While absolute rates are highly dependent on the list of mutations provided, the percent inhibition that leads to maximum of *R* appears to be independent of the actual mutations.

Two interacting effects can be seen in Fig. 2.

1. As drug doses increase, the fitness advantage of resistant mutants grows (Fig. 2a). This increases the rate of evolution as resistance mutations are more likely to be fixed (of note, these results are derived with the Moran model [42], with the additional assumption that deleterious mutations cannot become fixed). Because of the large population size (Table 1) and since in the small set of mutations considered there are few deleterious mutations, their contribution is negligible. If we assume a constant reproduction and mutation rate, the rate of evolution depends only on the probability of fixation for all the relevant SNVs. Mutations are rare enough that usually no more than one (relevant) SNV is present in the population at one time. Thus the rate of evolution for any mutation is approximately proportional to the sum of each individual SNVs fixation probability in a population of wildtype cells
  7 $$ R \propto \sum_{i \in \text{SNVs}}{ f_{i}H(f_{i})} \quad \text{where} \quad f_{i} = 1 - { 2^{C/{IC}_{50}^{(i)}} \over 2^{C/{IC}_{50}^{(wt)}} }   $$
  which, given these assumptions, is directly proportional to the rate of evolution. *H*(*x*) is the Heaviside step function:
  $$H(x) = \left\{ \begin{array}{ll} 0 & \quad x < 0 \\ 1 & \quad x \geq 0 \end{array} \right. $$
2. As drug doses increase, the overall reproduction rate slows down. Fewer cell divisions lead to fewer mutants which leads to a slower rate of evolution (Fig. 2c).

Unlike the reproduction rate, which can be slowed to an asymptotic halt, as drug doses go towards infinity fitness effects can never speed up evolution beyond a certain limit. With increasing selective advantage the probability of fixation of any resistance mutation, however weak, approaches 1. Thus the rate of evolution becomes entirely limited by how often resistance mutations occur. This results in the peaks evident in row b of Fig. 2, where at a medium degree of inhibition (slightly above 50%) reproduction rate is high enough to produce a significant number of mutants, and mutant fitness is high enough that the mutations are likely to be fixed once they occur. The rate of evolution then sharply decreases and a higher degree of inhibition results in a lower risk of resistance mutations, consistent with [43], who studied response to the degree of inhibition. At the lower inhibition end, this lends some extra credence to the idea of lowering doses for patients in major molecular response (MMR, a treatment response criterion based on very low levels of Bcr-Abl1 transcript) [17]. It seems that benefits might extend beyond lowering side effects into slowing down SNV-based resistance. It is however relatively well established [44] that higher doses create a more effective and lasting response. For initial treatment it is still likely that aiming for the highest achievable inhibition is the superior strategy even if it increases the risk of mutations. Apoptosis induced proliferation [3], where dying cells signal their neighbours to reproduce faster, can modify such models but is not considered here. Likewise, whether very low doses compare favourably to no treatment at all is not addressed by this model.

### TKI-rotation protocols

**Fig. 3:**
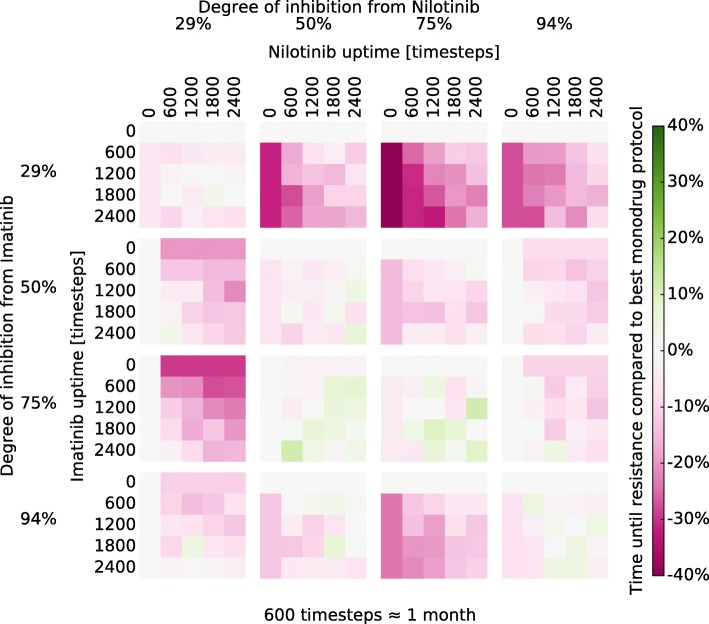
Relative change in $\text {WT}_{\frac {1}{2}}$ for Imatinib-Nilotinib rotation protocols. Each smaller heatmap shows the effects of different rotation timings at a certain dose of each drug. For instance, the (600 Imatinib, 1200 Nilotinib) box in the (50%, 50%) heatmap shows the effect of a drug rotation with *t*_*a*_=600 timesteps of Imatinib treatment (ca. 1 month) followed by *t*_*b*_=1200 timesteps of Nilotinib treatment (ca. 2 months), see Fig. 1, with both drug concentrations set such that cell growth was slowed by 50%. In half the simulations the rotation started with Imatinib, and in the other half it started with Nilotinib. The colours correspond to the change in $\text {WT}_{\frac {1}{2}}$ compared to a constant Imatinib or Nilotinib, whichever is better, with the same degree of inhibition. A zero duration uptime of one drug implies a constant concentration of the other. Zero-zero uptime boxes are set as 0%, as they cannot be assessed since drug resistance almost never occurs without selective pressure from drugs

**Fig. 4:**
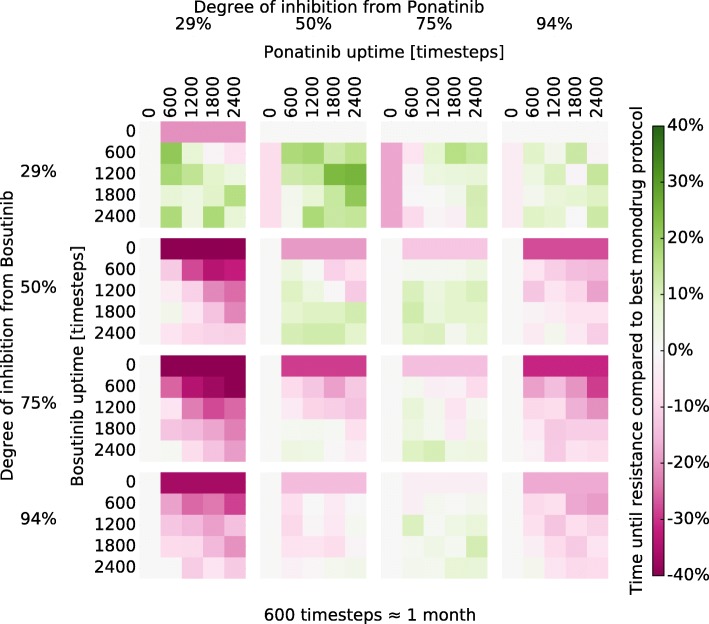
Relative change in $\text {WT}_{\frac {1}{2}}$ for Bosutinib-Ponatinib rotation protocols. See figure text of Fig. 3 for a detailed description of the plot layout

**Fig. 5:**
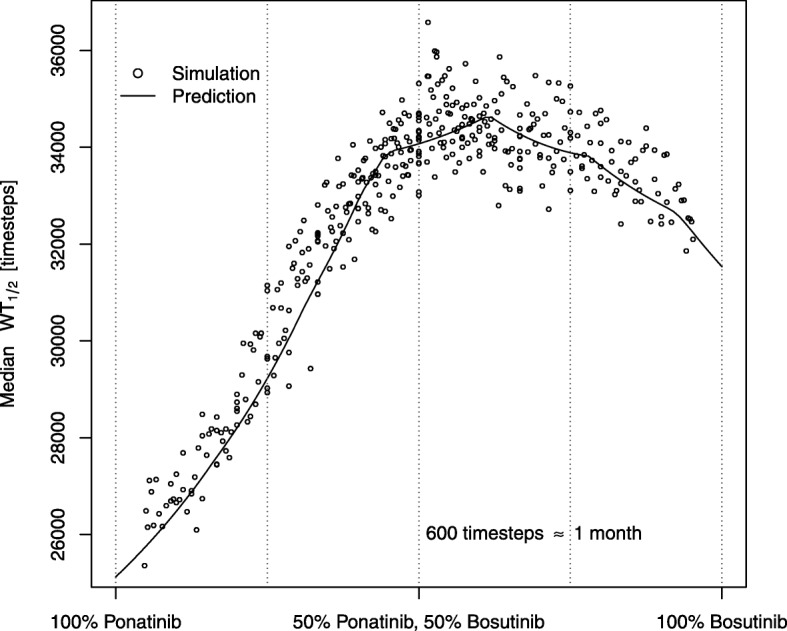
Expansion of the data presented in Figure 4, showing median $\text {WT}_{\frac {1}{2}}$ at 50% inhibition of both drugs as a function of the relative time each drug was used. The solid line is a theoretical prediction based on the table of drug sensitivity for each mutation, see Eq. (8)

TKI-rotation protocols with Imatinib-Nilotinib, and Bosutinib-Ponatinib were selected for examination. Our hypothesis was that drugs with more significant difference in their resistance mutation spectrum would be better candidates for rotation therapy. The treatment protocols were represented by interlaced square-waves of the two alternating drugs as shown in Fig. 1. These protocols were tested at a range of doses and timings as displayed in Figs. 3 and 4. Consistent with our hypothesis, there appears to be more potential benefits in Bosutinib-Ponatinib rotation, in contrast to Imatinib-Nilotinib rotation which appears roughly equivalent to constant protocols of either drug. The benefits are not universal to any dose-timing combination, though it appears possible to achieve a therapeutic benefit under a wide range of conditions. Introducing low dose (29%) Bosutinib always reduces the chance for resistance as compared with pure Ponatinib. However, such a concentration is probably too low to effectively treat active CML (prior to MMR). To a lesser degree, introducing a medium-high dose (75%) of Ponatinib also appears to be superior to pure Bosutinib in terms of resistance, but perhaps not side effects and toxicity. At 50% inhibition a Bosutinib-Ponatinib rotation slows the emergence of resistance by at best 7.5% or 36% over pure Bosutinib or Ponatinib respectively (Fig. 5). Thus, compared to switching drug upon resistance, a rotation treatment protocol would remain effective for about 4 months longer than the best constituent monodrug protocol. To examine the effect of a reduced population size, the simulations where repeated with $\hat {N} = 400$, as was used to model haematopoesis [31, 45]. No benefical effects were observed in that case (Section 9 and Figure S4 in Additional file 1). This could be because the increased randomness in when a mutation occurs obscures any effect. Another possibility is that cycle length was not optimal; in contrast to the large population where cycle length does not seem very important (see below) shorter fixation times in the small population might necessitate shorter cycles. However, as most estimates of the CSC populations size are almost three orders of magnitude larger than $\hat {N} = 400$ we did not examine this further.

Our results are consistent with recent clinical studies. A rotation of Bosutinib and Ponatinib, has been applied successfully in a multi-resistant patient [46], whereas a rotating protocol of Imatinib and Nilotinib was equivalent to standard therapy in its treatment effect [47]. Note also that even rotating protocols which do not have any significant improvement in the treatment effect still have benefits for side-effect management. The Bosutinib-Ponatinib rotation seems beneficial in tumours that exhibit wt Bcr-Abl under the right circumstances according to our model. Inhibition for a multitude of other combinations with varying degrees of potential benefit was also tested and the results are shown in Additional file 1: Figures S3a–S3j.

In general, the degree of inhibition and the timing ratios seem to be the biggest factors in determining the effect of a Bosutinib-Ponatinib rotation. The former is evident from Fig. 4. The latter is shown in Fig. 5 which demonstrates that protocol effects at a fixed degree of inhibition is mainly a function of the timing ratio. If we define *x* as the fraction of time dedicated to one of the drugs in a cycle (horizontal axis in Fig. 5), and we let

$$\psi_{i} = {1 - { 2^{-(1-x)C_{A}/{IC}_{50}^{(wt)} - {xC}_{B}/{IC}_{50}^{(wt)}} \over 2^{-(1-x)C_{A}/{IC}_{50}^{(i)} - {xC}_{B}/{IC}_{50}^{(i)}} }} $$

where *C*_*A*_ and *C*_*B*_ are concentrations of the respective drugs and the fitting parameters *k* and *m* are set such that the end-points match the $\text {WT}_{\frac {1}{2}}$ of pure Ponatinib or Bosutinib treatment, and

$$\phi_{i} = \psi_{i} H(\psi_{i}) $$

where *H*(*x*) is the Heaviside step function, then, the median $\text {WT}_{\frac {1}{2}}$ (Eq. 6) is approximately described by

8 $$ \text{WT}_{\frac{1}{2}}~(x) = {k \over \sum_{i \in {nn}_{wt}} \epsilon_{wt, i} \phi_{i}} + m   $$

Recall that *ε*_*ji*_ describes how likely a particular mutation is to occur (Eq. 4) and the summation is carried out for all residues that can be mutated (nearest neighbours, vide supra). Intuitively, $\text {WT}_{\frac {1}{2}}$ depends on the fixation probability of each of the possible mutations and how often they appear, all the while assuming fixation time is so much slower than drug rotation that the effects of two drugs can be incorporated. The fitness of any particular mutation will change with *x*; if it is resistant towards one drug but not the other then at some value of *x* it goes from being resistant to neutral and its fixation probability, *ϕ*_*i*_, goes from a finite positive number to zero. The full derivation is provided in Additional file 1: Section 6. Combining these effects from all possible mutations, and taking into account how often they appear (proportional to *ε*_*wt*,*i*_), results in Eq. 8 and the predictions shown in Fig. 5 and Figures S3a–S3f in Additional file 1. The irregular bumps in the curve occur when any particular mutation goes from being more resistant than the wildtype to being less resistant or vice versa; the large number of known mutations means that this happens several times for most drug combinations. No correlation could be found for cycle length within the tested intervals but some correlation must exist, since very long cycles approximate a constant protocol. Equation (8) is not valid for those cases as it assumes fixation time is much longer than cycle time.

The benefits of a rotation protocol are due to the positive (though small) probability that non-polyresistant mutants are exposed to a drug towards which they are sensitive before they can grow to become a significant part of the population. At best this eliminates them outright, but should that fail their growth can be slowed and replacement significantly delayed. Evolutionary studies suggest that when a new mutation occurs right before a drug switch, it is likely to be subsequently eliminated when the fitness landscape changes [48]. Another important effect of a shifting fitness landscape is a reduced effectiveness of selection. This is both beneficial and detrimental for this type of protocol design, for while it reduces the advantage of resistant clones it also makes it more unlikely for them to be eliminated.

**Fig. 6:**
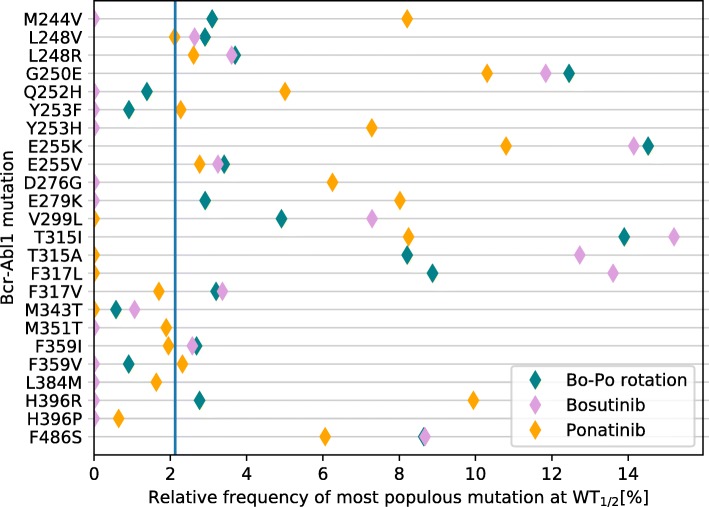
Distribution of most common mutation at $\text {WT}_{\frac {1}{2}}$ for three protocols, simulated 50 000 times each. The protocol has a significant effect on mutant distribution (*χ*^2^-test, *χ*^2^=56929, *p*<10^−16^). The vertical blue line shows the expected frequency assuming all mutations were equally likely and equally fit. Unobserved mutations and double (or higher) mutants are not shown. For these simulations, doses were set such that Bosutinib caused 50% inhibition and Ponatinib caused 75% inhibition. The simulation protocol was 3:1 Bosutinib:Ponatinib, i.e., Bosutinib was used during 1800 timesteps (ca. 3 months), followed by Ponatinib during 600 timesteps (ca. 1 month) etc

TKI-rotation protocols also seem to have an effect on the resulting distribution of observed mutations. Monodrug protocols of Bosutinib and Ponatinib and a drug rotation were simulated until a mutant had taken over in each simulation, and the most common mutation at $\text {WT}_{\frac {1}{2}}$ was recorded. The resulting mutant distribution is shown in Fig. 6. As is evident the rotation protocol can, somewhat counter-intuitively, cause any type of change in distribution, and is not limited to an interpolation between the two constant protocols. It is perhaps possible to exploit this such that evolution moves towards mutants where effective inhibitors are available. Whereas the example in Fig. 6 favours G250E and E255K, both of which are at least somewhat resistant to all available drugs, does not fall into this category, it is possible that some combinations would steer evolution towards more easily treatable mutations. For instance, if a highly effective drug existed against G250E and E255K which are made more common by the rotation in Fig. 6, the combination would effectively steer evolution towards a treatable set of resistance mutations with an increased probability. Indeed, if the onset of resistance cannot be effectively prevented or delayed, ensuring it happens in a less harmful way can still provide some benefit.

## Discussion

We have developed, implemented and tested a model for simulating treatment protocols based on available IC_50_ data. Using CML as an example we have shown that drug rotation therapy with available Bcr-Abl TKIs could potentially decrease the risk of resistance. These are some suggested guidelines as to when it may be better that standard therapy:

- The drugs have different resistance profiles.
- There is a sub-optimal drug response from a molecular point of view, i.e. the amount of inhibition achieved places the patient in the zone where resistance happens the fastest (ca. 50% inhibition, Fig. 2).

The first condition is fulfilled by different combinations of Abl1 inhibitors, e.g., Nilotinib + Dasatinib and Bosutinib + Ponatinib. As for the second condition, it is clear that all drugs reduce the number of tumour cells efficiently, but some persistent cells seem to survive (at least with Imatinib, which is the most common inhibitor). In addition, a rotation protocol may be useful if an inhibitor is working fine but leads to difficult side effects. Furthermore, the timing ratio appears to be more important than exact cycle length, so cycle length could be optimised for side effect reduction or other factors.

In developing the model, we chose to use a stable population size. This choice is based on the assumption that cancer stem cells, that drive the evolution within the tumour, are never eliminated by TKIs though their proliferation may be hindered. It should be noted, however that the results could be influenced by this assumption. Another necessary limitation of the model is that IC_50_ values are not always consistent. Measured IC_50_ values vary significantly between studies [49]. On the other hand, there is evidence that they have clinical relevance for drug selection [50]. Compound mutations are included in the model. IC_50_ values are available for those that are most clinically relevant, whereas in the other cases we made the assumption that the double mutant is as resistant as the most resistant single mutation. Yet another limitation is ignoring the effect of pharmacokinetics and treating drug doses as constant. Patients do not experience a truly constant drug dose in practice, and it is known to have an effect under other circumstances [51, 52]. Investigating the effects of this is a potential direction for future studies.

## Conclusions

The potential delay in the onset of resistance has to be weighed against the risk of more severe side-effects. The greatest gains predicted by our model occur with rotations involving Ponatinib. Whereas a drug applied intermittently in a drug rotation is likely more well tolerated than if taken continuously, it seems unlikely that benefits would outweigh the risk for rotations involving Ponatinib. However, having shown that the potential could exist we recommend considering drug rotations if more well tolerated options are developed.

While no other malignancy fits this model quite as well as CML, there are similar resistance mutation phenomena in Gastrointestinal stromal tumour (GIST), Ph ^+^ acute lymphoblastic leukaemia (ALL), EGFR-mutant non small cell lung cancer (NSCLC), ALK-rearranged NSCLC, and other cancers. Ph ^+^-ALL has the same molecular driver and develops resistance in a rather similar way [53]. In GIST, which is also treated with Imatinib, 50%-70% of late progression cases were suggested to be caused by mutations which affect drug binding interactions [54]. Both variants of NSCLC are also affected to some extent by kinase domain mutation induced resistance, though it accounts for a smaller fraction of observed cases [55, 56]. For the last three, the spatial structure of solid tumours might also limit the applicability of this model, which does not include local competition effects, and/or differential exposure to drugs. We note though that these two are having the opposite influence, i.e., cells that out-compete others for development because they have better exposure to nutrients are also more accessible to the drugs, and hence the conclusions may be valid even for solid tumours.

## Additional file

Additional file 1 Derivations of formulae, implementation details, justification of assumptions and figures too large for the main text. (PDF 277 kb)

## Abbreviations

ABL1: Abelson tyrosine kinase 1
ALK: Anaplastic lymphoma kinase
ALL: Acute lymphoblastic leukaemia
BCR: Breakpoint cluster region
CML: Chronic myeloid leukaemia
CSC: Cancer stem cell
EGFR: Epidermal growth factor receptor
GIST: Gastrointestinal stromal tumour
MMR: Major molecular response
NSCLC: Non small cell lung cancer
Ph ^+^: Philadelphia chromosome positive
PSSM: Position specific scoring matrix
SNV: Single nucleotide variation
TKI: Tyrosine kinase inhibitor
