# Supplementary material for: Stochastic modelling of tyrosine kinase inhibitor rotation therapy in chronic myeloid leukaemia
Source: BMC Cancer. 2019 May 28;19:508. doi: 10.1186/s12885-019-5690-5 (PMC6540367; doi:10.1186/s12885-019-5690-5)
Supplement: Supplementary file 1 — Derivations of formulae, implementation details, justification of assumptions and figures too large for the main text. (PDF 277 kb) [file 12885_2019_5690_MOESM1_ESM.pdf]

Supplementary information for: Stochastic modelling of tyrosine kinase inhibitor rotation therapy in chronic myeloid leukaemia

1 Computing reproduction rates from PSSM data

CML cells depend on the ability of Abl to catalyse the phosphorylation of downstream effectors and thereby promote tumour progression. It has been shown that mutants that lead to a higher catalytic efficiency are also associated with higher growth rates [1]. Under the assumption that enzymes evolve for catalytic efficiency [2], we can estimate the effect of mutating specific residues in the enzyme by how conserved they are throughout evolution, assuming that any such changes do not affect the specificity of the enzyme. Positions specific scoring matrices (PSSMs) list a $\log_2$-odds chance of finding a specific residue at a specific position in a protein. Using a PSSM derived from homological proteins in evolutionarily distant relatives, and assuming that the frequency with which specific mutations are found reflects their effects on the individuals fitness it becomes possible to find the reproduction rate these mutants would need to eventually establish the same frequency in a large population under our model.

Forgoing normalization, the expected population size of a length 1 protein is simply

$$2^a$$

given PSSM score a. We can ignore normalization, since the expected size is measured in comparison to expected sizes of all other amino acid variants at that position. For a full protein, the expected population size becomes

$$\hat{p}_i = \prod_k 2^{a_k}$$

multiplying over every position k in the protein specified by an amino-acid sequence i. This assumes that the effects of all changes are independent which, while obviously false, is justifiable assuming that larger more unpredictable exceptions usually require several mutations. A single mutation is independent by definition, and double or (rare) triple mutations are still small enough changes to the protein that they might be approximately independent most of the time. For higher order mutations the assumption almost surely breaks down more frequently, but these are practically irrelevant so the inaccuracies have no actual effect on clinically relevant simulations where more than a double mutant is unlikely and inadvisable. We can then find model parameters that create the same expected population size. In our model, the changes in population of every cell phenotype is described by the equation

$$N_i(t) = [1 + s_i(1 - \mu_i)](1 - q(t-1))N_i(t-1) + \sum_{j \in nn_i} s_j \mu \epsilon_{ji} N_j(t-1)[1 - q(t-1)]$$

which has been explained in more detail in the main article. From this equation we can determine the reproduction rates s_i that result in predetermined average population sizes for all genotypes, $\hat{p}_i$ by an iterative calculation

$$s_i^{(0)} = 0$$

$$s_i^{(n)} = \frac{q}{1 - \mu_i} - \sum_{j \in nn_i} s_j^{(n-1)} \frac{\epsilon_{ji}}{(1 - \epsilon_{ji})} \frac{\hat{p}_j}{\hat{p}_i}$$

until $s_i^{(n)}$ converges. Convergence happens relatively quickly ($n \approx 20$ is enough for `double` precision) for most inputs (although convergence is not guaranteed). The naive implementation calculating every s_i separately quickly runs into scaling issues, as branching of the nearest neighbours creates a large dependency tree. However, as $s_i^{(n)}$ depends only on $s_j^{(n-1)}$ by calculating $s_i^{(n)}$ for all i simultaneously and storing them for the next iteration, all of the branching is removed. This way, it can be done reasonably quickly even for very large sets of possible phenotypes.

The differences in reproduction rate derived with this model are generally very small (<0.1%)

2 Software details

The main simulation software is written in C++14 and can make use of local parallelism. Input file construction and output file analysis is performed by a set of python3 programs. The simulation time can be altered significantly by scaling the input parameters, but effects outside of small variations have not been examined. For comparison, the experiment presented in Figure 4 of the main text, comprising 400 000 individual simulations, finished in about 250 core hours running on an Intel Xeon E5-2630 CPU. Scaling was tested on up to 30 cores and is almost linear. Simple facilities exist for splitting an input file for utilising multiple machines, but no built in multi-node support has been implemented.

3 Rate of evolution when all mutations are guaranteed to be fixed

Given the relationship between growth rate and drug dose

$$s_i(t) = s_i^{(0)} 2^{-C(t)/IC_{50}}$$

and the assumption that all mutations become fixed (i.e. they have an infinite fitness advantage), the rate of evolution is directly proportional to the growth rate.

$$R \propto 2^{-C(t)/IC_{50}}$$

The degree of inhibition is

$$x = 1 - 2^{-C(t)/IC_{50}}$$

substitute into the equation before it and we get

$$R \propto (1 - x)$$

producing the straight lines seen in Figure 2C of the main text. The different slopes depend on what resistance mutations are possible for each drug.

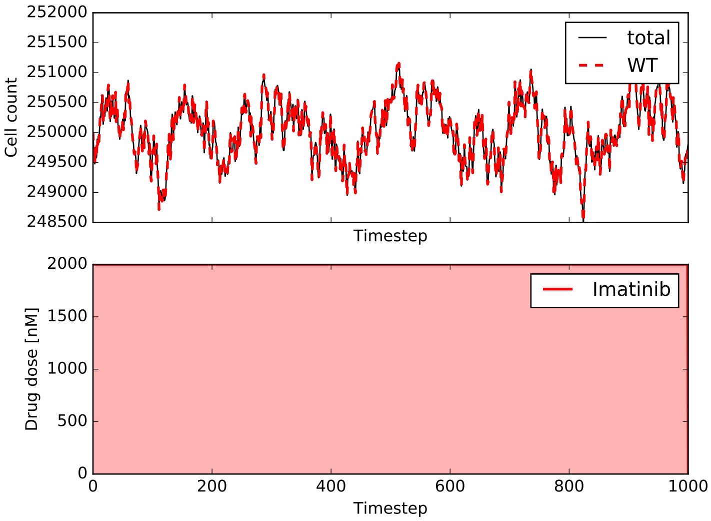

Figure 1: Example of population size control under a constant Imatinib treatment. As the fluctuations are relatively rapid, they are clearly visible even in a short simulation; in this case no resistance mutation had time to occur during the simulation period.

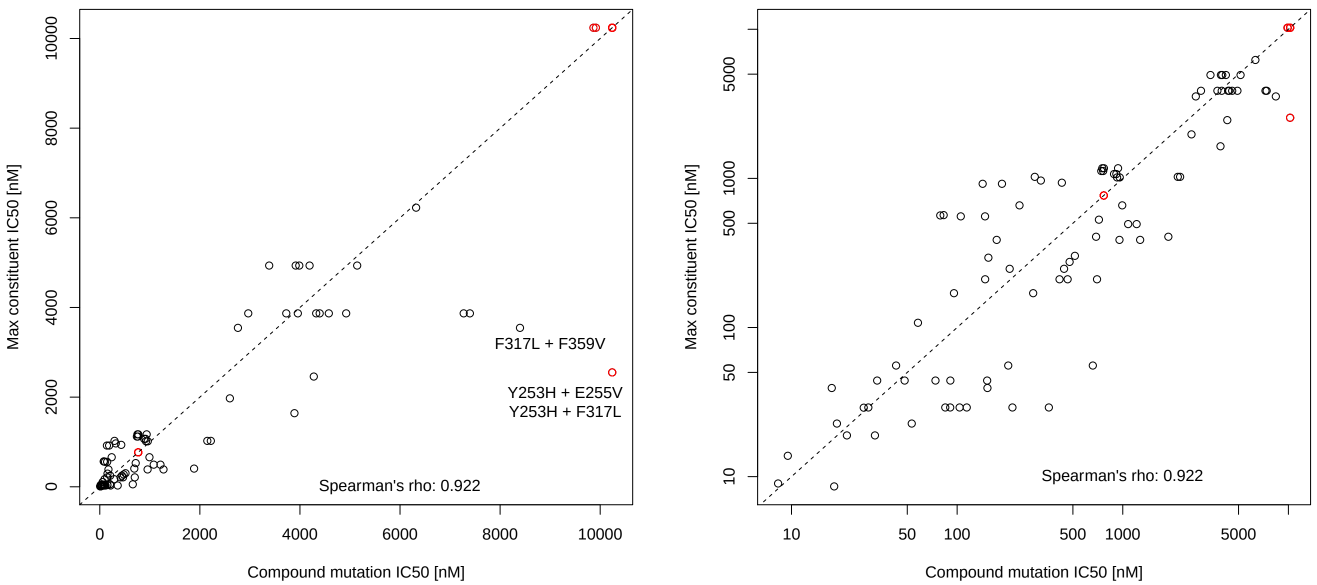

Figure 2: Plots comparing the correlation between the IC_{50} of the most resistant constituent SNV of a compound mutation, and the IC_{50} of the compound mutation. Left and right plots show linear and log-log plot of the same data respectively. For the red points at least one of the IC_{50} values is a minimum, rather than an exact value. The dashed line shows a 1:1 correlation. Data from [3, 4]

4 On IC_{50} values and Equation (1)

IC_{50} values are the main way BCR-ABL1 mutant drug sensitivity is reported. Generally these are measured by allowing cells to grow at a range of inhibitor concentrations for a fixed amount of time, then measuring cell proliferation using either a [^{3}H]-Thymidine incorporation assay or a tetrazolium based assay (such as MTS or MTT based assays). These assays measure values proportional to the number of living cells in the culture. Depending on how these results are analysed, the IC_{50} values reported could be telling us either

1. the dose required to reduce growth rate to ½ of the uninhibited rate or
2. the dose required to reduce the number of cells (as measured with one of the aforementioned assays), after a growth period t_e, to ½ the uninhibited number.

We refer to the IC_{50} values of case 1 ${IC_{50}}^{(1)}$ and of case 2 ${IC_{50}}^{(2)}$. If we consider Equation (2) in the main paper as a description of how drug doses impact reproduction rate (case 1), then the number of cells over time is described by

$$N(t) = N_0 2^{s^{(0)} t 2^{-C/IC_{50}^{(1)}}}$$

assuming cell density is low enough that exponential growth is an adequate description. Then, the count based ${IC_{50}}^{(2)}$ is the concentration where at some time t_e the cell count is the average of N_0 and $N(t_e)$

$$\frac{N_0 2^{s^{(0)} t_e} + N_0}{2} = N_0 2^{s^{(0)} t_e 2^{-IC_{50}^{(2)}/IC_{50}^{(1)}}}$$

which rearranges to

$$\frac{IC_{50}^{(2)}}{IC_{50}^{(1)}} = -\log_2\left(\frac{1}{s_0 t_e}\log_2\left[\frac{2^{s_0 t_e}+1}{2}\right]\right)$$

the right hand side of which is simply a constant. The two ways of processing the data thus yields proportional results. Because we define doses by the degree of inhibition they create, i.e. relative to the IC_{50} values, the precise nature of how they were determined can thus be ignored as the relative inhibition between different mutations is preserved regardless and the proportionality constant will be implicitly baked into the chosen concentrations.

5 Mutation rates

Because of redundancy in the genetic code, and because transitions are more common SNVs than transversions are mutation rates have to be calculated individually for each mutation.

	A	T	C	G
A	1	1	1	4
T	1	1	4	1
C	1	4	1	1
G	4	1	1	1

For instance, the mutation rate for ACT → ATT (most common T315I) is $\mu * 4$. In the program the (relevant) genome is described exactly and this is the method used to find mutation chances. However, when phenotypes are considered rather than genotypes (which is what matters from a resistance point of view) redundancy of the genetic code sometimes increases the mutation rate further. Consider a situation where a TTC phenylalanine mutating to a leucine causes resistance. This can happen in three ways via SNV: TTC → TTA, TTG, CTC. So the odds of the phenotype mutation is the sum of all three genotype specific mutation probabilities $\mu*(1+1+4)$. The mutation rates of all commonly included codons in the simulations in the paper are included below.

Synonymous mutations are also allowed. In the phenylalanine to leucine example above we could see TTC → TTT ($\mu*4$). From there, it could mutate into leucine via TTT → TTA, TTG, CTC ($\mu*(1+1+4)$). Such a sequence is unlikely but possible. Since synonymous mutations are allowed, it is possible that options outside of the table below could occur.

Table 1: Mutation rate modifiers for most common codons in simulations from this paper.

Mutation	Starting codon	Ending codon(s)	Probability Modifier (ϵ_{ji})
M244V	ATG	GTG	4
L248R	CTG	CGG	1
L248V	CTG	GTG	1
G250E	GGG	GAG	4
Q252H	CAG	CAC	1
Y253F	TAC	TTC	1
Y253H	TAC	CAC	4
E255K	GAG	AAG	4
E255V	GAG	GTG	1
D276G	GAC	GGC	4
E279K	GAG	AAG	4
V299L	GTG	CTG, TTG	2
T315A	ACT	GCT	4
T315I	ACT	ATT	4
F317L	TTC	TTA, TTG, CTC	6
F317V	TTC	GTC	1
M343T	ATG	ACG	4
M351T	ATG	ACG	4
F359I	TTC	ATC	1
F359V	TTC	GTC	1
L384M	CTG	ATG	1
H396P	CAT	CCT	1
H396R	CAT	CGT	4
F486S	TTT	TCT	4

6 Derivation of equation (8)

The time between the start of the simulation and the first mutation becoming fixed consists of two parts: the time until the mutation that will become fixed appears, and the time it takes for the mutated population to become dominant (the fixation time). However, we cannot directly calculate the median time until some mutation becomes fixed, as we do not know the distribution of the fixation times. We therefore estimate the median based on how these two different intervals depend on growth and mutation rates, leaving some fit parameters.

To derive equation (5) we start by noting that the typical fixation time of a mutation is much longer than the time interval over which the drug treatments are rotated. As a result of this, we may approximate the growth rate of a given mutated population $r^{(i)}$ as time-independent and equal to the average over the interval, i.e.,

$$\frac{r^{(i)}}{\ln 2} = (1-x)\frac{C_A}{IC_{50}^{(i)}} + x\frac{C_B}{IC_{50}^{(i)}} = r_2^{(i)} \ .$$

This expression is accurate up to next-to-leading order in the ratio of the rotation interval and typical fixation time. The growth rate determines the fixation probability, which can be obtained based on the Moran model, as described in the main manuscript,

$$\phi_i = 1 - \frac{2^{r_2^{(wt)}}}{2^{r_2^{(i)}}} \ .$$

Until one of the mutations dominates, the population consists mostly of wt cells. Hence, the fixation rate of a specific mutation is proportional to the rate at which the wt cells can mutate in this way, multiplied by the fixation probability, i.e. $\epsilon_{wt}\phi_i$. The total fixation rate can thus be approximated as

$$\mu\bar{\phi} = \mu \sum_{i\in nn_{wt}} \epsilon_{wt}\phi_i \ .$$

The typical time it takes for a mutation to occur that will eventually be fixed is then $\propto 1/\bar{\phi}$. Once a mutation that will be fixed has occurred, it will take some more time for it to actually become dominant. We can estimate the typical fixation time for each separate mutation by the inverse of the growth rate. This rate is much higher than the rate at which mutations appear, and we may therefore estimate the typical fixation time with a next-to-leading order term as a constant. The time of the entire event, from the start, the mutation stage, and finally until fixation of some mutation, should then typically behave as

$$WT_{1/2}\ (x) = \frac{k}{\bar{\phi}} + m \ .$$

7 Prediction versus simulation of all (approved) drug rotations (Figure 3)

As can be seen in the figures below, the approximate model described by equation (8) in the main paper predicts with reasonable accuracy the simulation results for rotations with any pair of approved drugs. The solid lines are fit using only the endpoints (i.e. $WT_{1/2}$ of a pure single-drug protocol), and the dashed lines use a least-squares fit. In most cases fitting based on only the end points is satisfactory, with the exception of Imatinib-Nilotinib, Imatinib-Dasatinib and Imatinib-Ponatinib rotation (Figures (3a), (3b) and (3d)). In those cases an inconsistency between the predicted resistance rates for pure protocols and the simulated rates lead to inaccurate fitting. A least-squares fit works better for those cases. Rotations involving Ponatinib (Figures (3d), (3g) ,(3i) and, (3j)) result in the biggest benefits when compared to pure protocols of either drug involved.

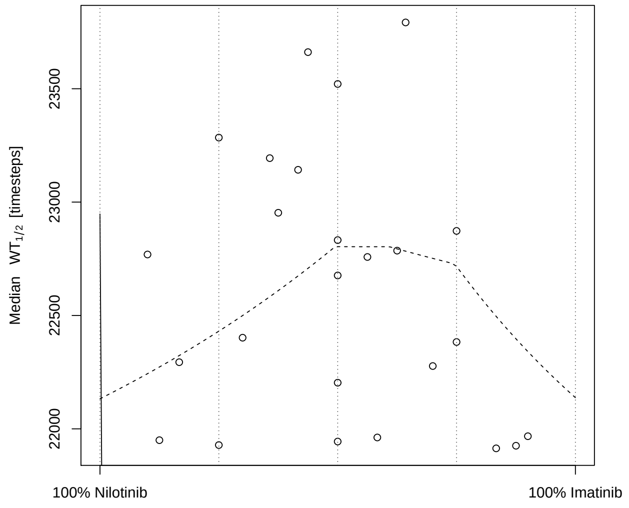

(a)

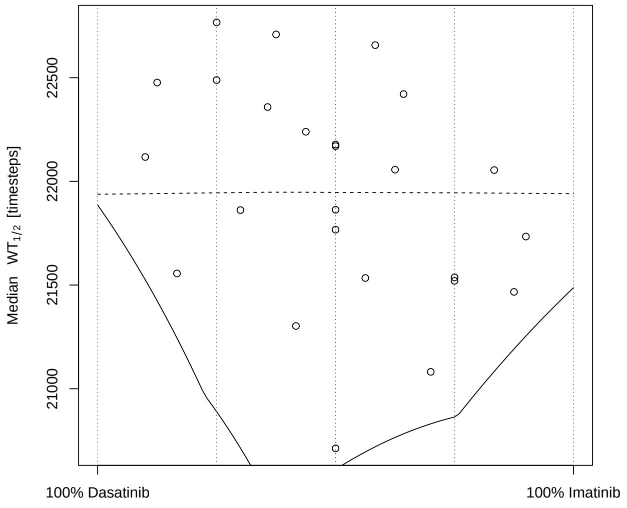

(b)

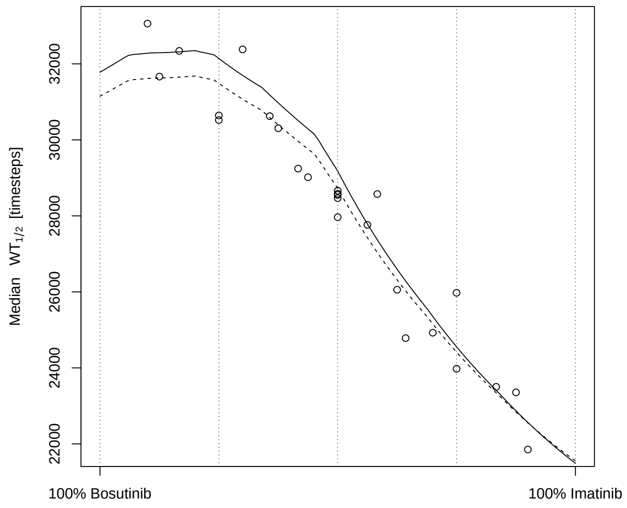
Median WT1/2 [timesteps]
22000
24000
26000
28000
30000
32000
100% Bosutinib
100% Imatinib

(c)

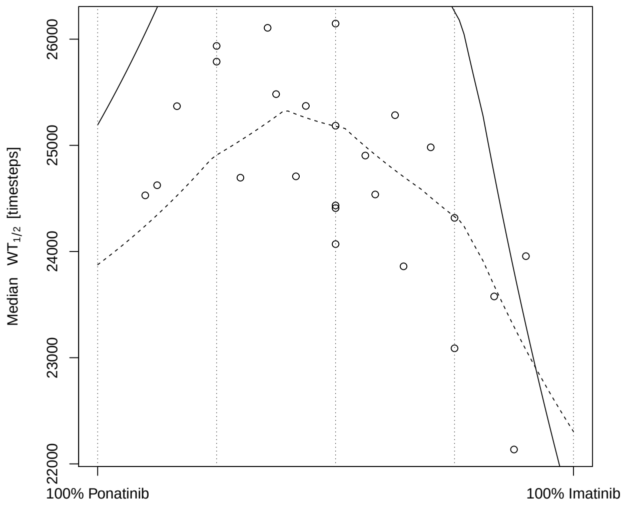
Median WT1/2 [timesteps]
22000
23000
24000
25000
26000
100% Ponatinib
100% Imatinib

(d)

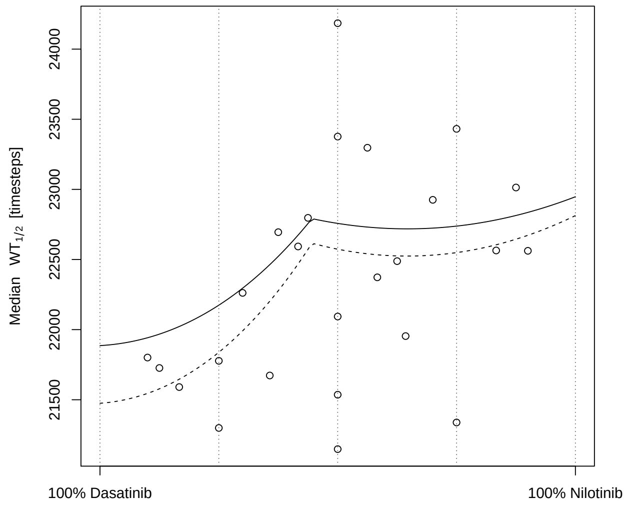
Median WT1/2 [timesteps]
21500
22000
22500
23000
23500
24000
100% Dasatinib
100% Nilotinib

(e)

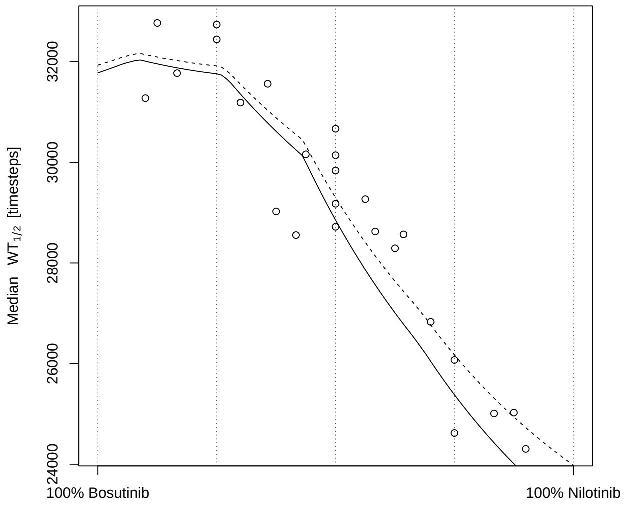
Median WT1/2 [timesteps]
24000
26000
28000
30000
32000
100% Bosutinib
100% Nilotinib

(f)

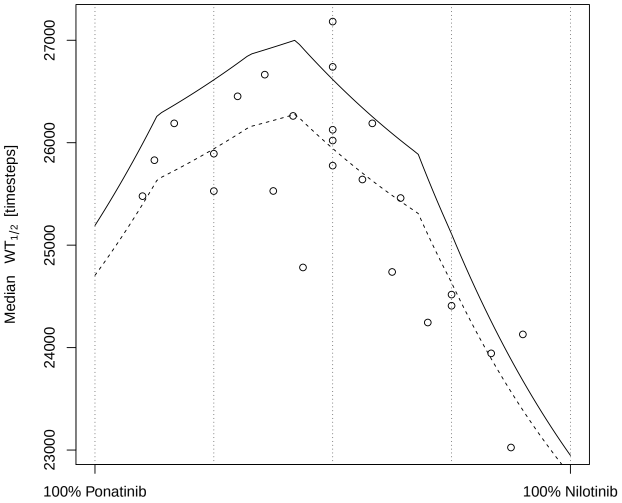
Median $WT_{1/2}$ [timesteps]
27000
26000
25000
24000
23000
100% Ponatinib
100% Nilotinib

(g)

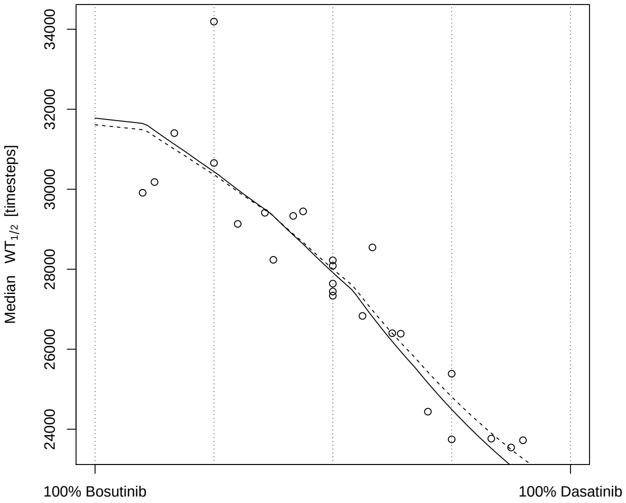
Median $WT_{1/2}$ [timesteps]
34000
32000
30000
28000
26000
24000
100% Bosutinib
100% Dasatinib

(h)

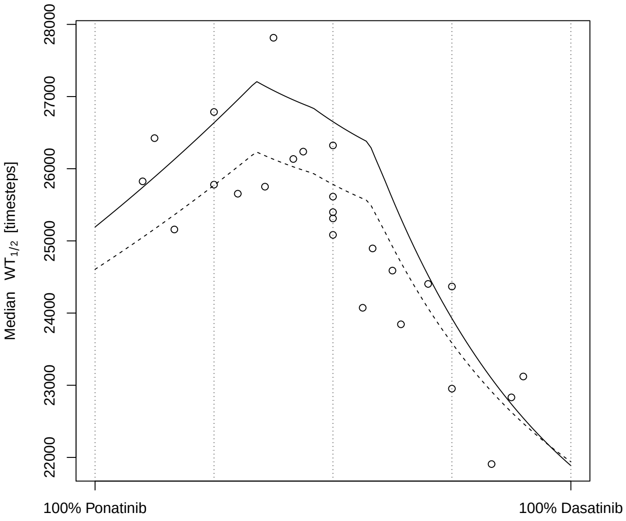
Median $WT_{1/2}$ [timesteps]
28000
27000
26000
25000
24000
23000
22000
100% Ponatinib
100% Dasatinib

(i)

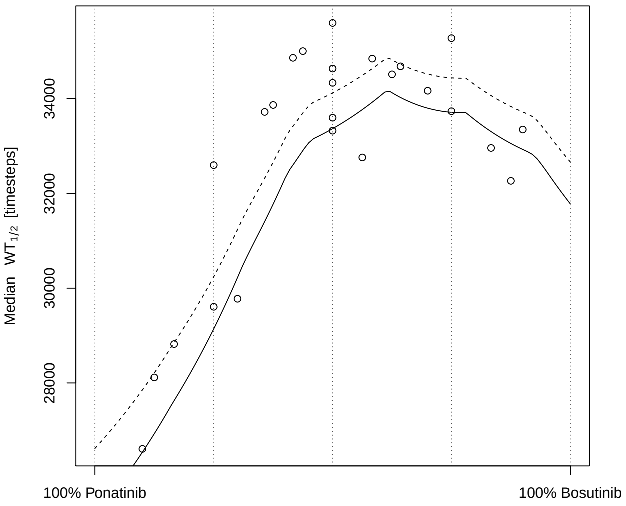
Median $WT_{1/2}$ [timesteps]
34000
32000
30000
28000
100% Ponatinib
100% Bosutinib

(j)

8 Algorithm pseudocode

1: $P \leftarrow$ Dictionary from genes i to cell counts N_i
2: **repeat**
3: Rebalance death rate, q
4: **for all** Gene and cell count pairs $(i, N_i) \in P$ **do**
5: $N_i \leftarrow N_i - B(N_i, q)$
6: **end for**
7: Remove any gene-cell count pairs from P where $N_i \leq 0$
8: $P_{\text{new}} \leftarrow$ Empty dictionary
9: **for all** $(i, N_i) \in P$ **do**
10: $n \leftarrow B(N_i, s_i(t))$
11: $m \leftarrow B(n, \mu_i)$
12: $N_i \leftarrow N_i + n - m$
13: **for** each new mutant (m repeats) **do**
14: Mutated gene $i^\dagger \leftarrow$ Mutate(i)
15: $P_{\text{new}}[i^\dagger] \leftarrow 1$
16: **end for**
17: **end for**
18: $P \leftarrow$ Merge(P and P_{new})
19: **until** set number of iterations, or other end condition

Algorithm 1: Model of stochastic cell growth. Initial population set on line 1 is defined by the input file. On line 15 and 18 if a gene already exists in the dictionary, the cell counts are added together.

9 Small population size

A drug rotation of Imatinib and Ponaitnib was chosen to test the whether the observed effects are present at a drastically reduced population size ($\hat{N} = 400$) as it has the greatest potential benefit according to theory (Supplementary material Section 7). A cycle time of 1200 timesteps per drug was used and simulation time was extended significantly, see Figure 4. As a consequence of the small population size, the observed values of $\mathrm{WT}_{1/2}$ were much larger than those reported for a simulation with 200,000 cells; since the mutation rate and division rate were maintained it is very unlikely for mutations to occur. However, increasing mutation or division rate does not seem to be in line with the biological reality, since 10^{-7} is already a higher-end mutation rate, and the most basal stem cells that the reduced pool is intended to model divide very slowly

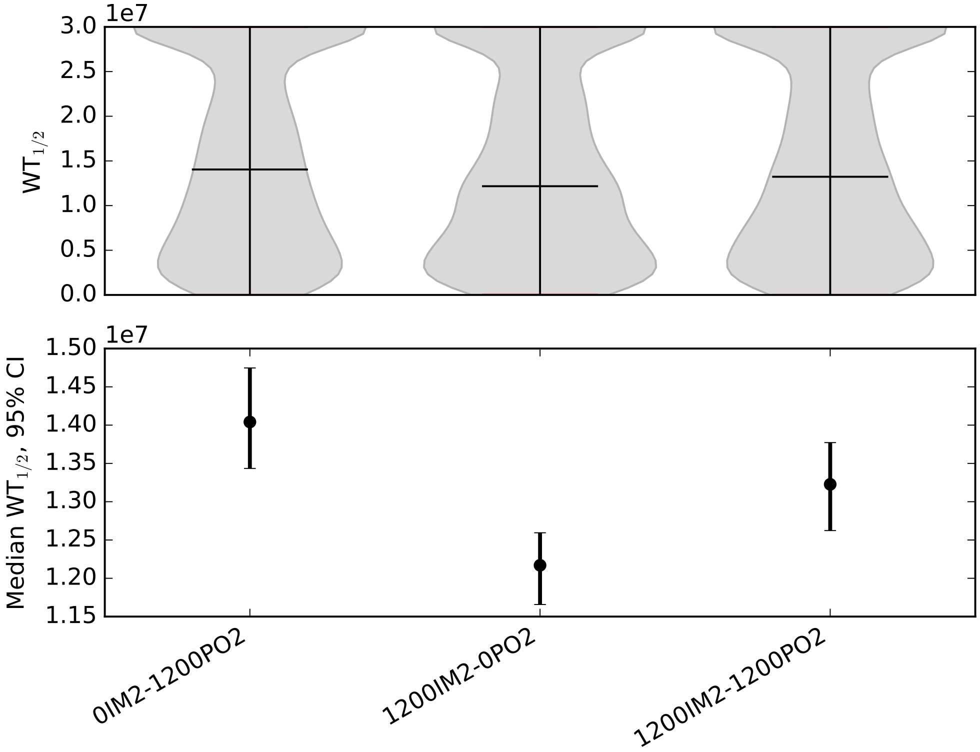

Figure 4: Comparison of a 2 months Imatinib, 2 months Ponatinib drug rotation protocol with monodrug versions in a 400 cell population. Each protocols was simulated 4000 times. The top panel shows a violin plot of the $WT_{1/2}$ values from the simulation, with grey area showing probability density and horizontal black lines showing medians. The bottom panel shows the median $WT_{1/2}$ for each protocol with a bootstrapped 95% confidence interval. The expected result was a higher $WT_{1/2}$ for the drug rotation (1200IM2-1200PO2) than for either monodrug protocol, as in Figure 3d (section 7) above, however, the trend seems to be simply linear between the pure Imatinib (1200IM2-0PO2) and pure Ponatinib (0IM2-1200PO2).

References

[1] Griswold, I., MacPartlin, M., Bumm, T., Goss, V., O'Hare, T., Lee, K., Corbin, A., Stoffregen, E., Smith, C., Johnson, K., Moseson, E., Wood, L., Polakiewicz, R., Druker, B., Deininger, M.: Kinase domain mutants of bcr-abl exhibit altered transformation potency, kinase activity, and substrate utilization, irrespective of sensitivity to imatinib. Mol. Cell. Biol. **26**(16), 6082–6093 (2006). doi:10.1128/MCB.02202-05

[2] Albery, W.J., Knowles, J.R.: Evolution of enzyme function and the development of catalytic efficiency. Biochemistry (Mosc.) **15**(25), 5631–5640 (1976). doi:10.1021/bi00670a032. https://doi.org/10.1021/bi00670a032

[3] Zabriskie, M.S., Eide, C.A., Tantravahi, S.K., Vellore, N.A., Estrada, J., Nicolini, F.E., Khoury, H.J., Larson, R.A., Konopleva, M., Cortes, J.E., Kantarjian, H., Jabbour, E.J., Kornblau, S.M., Lipton, J.H., Rea, D., Stenke, L., Barbany, G., Lange, T., Hernndez-Boluda, J.-C., Ossenkoppele, G.J., Press, R.D., Chuah, C., Goldberg, S.L., Wetzler, M., Mahon, F.-X., Etienne, G., Baccarani, M., Soverini, S., Rosti, G., Rousselot, P., Friedman, R., Deininger, M., Reynolds, K.R., Heaton, W.L., Eiring, A.M., Pomicter, A.D., Khorashad, J.S., Kelley, T.W., Baron, R., Druker, B.J., Deininger, M.W., OHare, T.: BCR-ABL1 compound mutations combining key kinase domain positions confer clinical resistance to ponatinib in Ph chromosome-positive leukemia. Cancer Cell **26**(3), 428–442 (2014). doi:10.1016/j.ccr.2014.07.006

[4] Zabriskie, M., A Eide, C., Yan, D., Vellore, N., D Pomicter, A., L Savage, S., J Druker, B., W Deininger, M., O'Hare, T.: Extreme mutational selectivity of axitinib limits its potential use as a targeted therapeutic for BCR-ABL1-positive leukemia. Leukemia **30** (2015)
